# Pioneering Perianesthesia Nursing in Japan: A Mixed-Methods Evaluation of Participant Satisfaction and Current Strategic Perspectives

**DOI:** 10.7759/cureus.106162

**Published:** 2026-03-30

**Authors:** Yuko Akanuma

**Affiliations:** 1 Nursing, International University of Health and Welfare, Narita, JPN

**Keywords:** advanced practice nurse, nurse anesthetists, nurse practitioner (np), perianesthesia nursing, perioperative nursing

## Abstract

Introduction: Perianesthesia nursing is an emerging specialty in Japan, and its educational framework and professional roles are still developing. In 2010, our graduate school established the first educational program for perianesthesia nurses (PANs). To support continuing education and professional networking, an annual study meeting has been held for healthcare professionals involved in perioperative care. This study aimed to evaluate participants’ satisfaction with the educational sessions and to identify challenges and future directions for perianesthesia nursing education in Japan.

Methods: A questionnaire survey was conducted among participants of a perianesthesia nursing study meeting held outside the institution in 2019. A self-administered questionnaire consisting of 20 items and open-ended questions was distributed to 100 participants. Satisfaction with educational lectures and program management was assessed using Likert-scale items. Descriptive statistics and correlation coefficients were calculated, and qualitative content analysis was conducted on free-text comments.

Results: Sixty-three valid responses were obtained (response rate 63%). The majority of respondents reported being “extremely satisfied” or “somewhat satisfied” with the educational sessions. The keynote lecture on anesthesiology education received the highest rating. Overall satisfaction with program management was high, despite comparatively lower ratings for time allocation. Content analysis identified three categories: proposals for multidisciplinary exchange programs, insufficient understanding of PAN activities, and questions regarding the professional significance of PANs.

Conclusions: The survey revealed that nurses attending the perianesthesia study sessions were largely experienced and expressed high overall satisfaction with both the educational content and the session management. Despite this positive feedback, participants sought greater opportunities for multidisciplinary interaction and clearer insight into the evolving role of PANs. Given that nurse anesthetists’ autonomy and scope of practice vary widely across countries but generally trend toward advanced, independent practice, the Japanese perianesthesia nursing role must be redefined and its career pathways strengthened to align with international advanced practice standards.

## Introduction

Perioperative care requires coordinated teamwork among surgeons, anesthesiologists, nurses, and other healthcare professionals to ensure patient safety and optimal surgical outcomes. In many countries, advanced practice nurses play essential roles in perioperative and anesthesia-related care [1]. For example, nurse anesthetists and advanced practice nurses are involved in perioperative patient management, anesthesia assistance, patient education, and quality improvement initiatives [2]. Studies have shown that the integration of advanced practice nurses into perioperative teams contributes to improved patient safety and efficiency of care delivery.

In particular, certified registered nurse anesthetists (CRNAs) in the United States have a long history of providing anesthesia care and perioperative support. Their role has been recognized as an important component of the anesthesia workforce, especially in addressing shortages of anesthesiologists and improving access to surgical services [3]. Similarly, advanced practice nurses in perioperative care settings have expanded responsibilities in patient assessment, perioperative management, and interprofessional collaboration [2,4].

In Japan, however, the development of specialized nursing roles in anesthesiology and perioperative care remains limited. Perianesthesia nursing has only recently begun to emerge as a specialized field of nursing practice. In 2010, a graduate-level program for perianesthesia nurses (PANs) was established at our institution to educate nurses with advanced competencies in anesthesia-related patient care [5].

Although the number of trained PANs has gradually increased, the professional role and academic framework of perianesthesia nursing have not yet been fully established in Japan. Furthermore, awareness of the PAN role among healthcare professionals remains limited. To promote continuing education and facilitate professional networking, an annual study meeting on perianesthesia nursing has been organized for healthcare professionals interested in perioperative care [4].

Evaluating participants’ experiences and satisfaction with such educational initiatives is essential for improving continuing education programs and for supporting the development of emerging nursing specialties. While the primary data were collected in 2019 and no subsequent longitudinal survey was conducted, this dataset has acquired renewed significance. The rapid evolution of nursing roles, driven by national labor reforms and the acceleration of task-shifting from physicians, makes the findings of this survey critically relevant to the current discourse on nursing professionalization in Japan. Therefore, the purpose of this study was to evaluate participant satisfaction with the educational sessions and to identify the current challenges and future directions for the role of advanced practice nurses in Japan.

## Materials and methods

This study employed a mixed-methods design combining a questionnaire survey and content analysis. Data were collected in February 2019 during a perioperative care nursing program session held outside a hospital setting. The study participants consisted of 100 healthcare professionals who attended the program. A self-administered questionnaire was distributed to participants at the end of the session. Participants were informed of the study objectives, the confidentiality of the data, and the voluntary and anonymous nature of participation.

The questionnaire consisted of 20 items, including participant characteristics (years of clinical experience, affiliation, and professional role), satisfaction with individual educational lectures, and evaluation of the quality of program management. Satisfaction was assessed using a four-point Likert scale. In addition, open-ended questions were included to obtain feedback on educational content and suggestions for future improvement (Appendix). The questionnaire was developed by the author with three colleagues to assess content validity and ease of response. Based on their feedback, minor revisions were made prior to finalization. For the quantitative analysis, descriptive statistics were used to summarize participant characteristics. Additionally, non-parametric correlation coefficients were calculated to examine the relationship between specific evaluation items and overall satisfaction regarding both the planning and educational content. Regarding the qualitative analysis, open-ended responses were analyzed to identify specific challenges related to participants' perceptions and needs. Key statements were coded, grouped into subcategories based on conceptual similarity, and further classified into categories to conceptualize the participants' perspectives.

This study was approved by the Institutional Review Board of St. Luke’s International University (Approval No. 18-R195). Although the study title was updated to reflect the current strategic perspective, the data collection procedures remained consistent with the approved protocol.

## Results

Participant characteristics

Sixty-three valid responses were obtained (return rate: 63%). The participants were predominantly working nurses, of whom half had over 10 years of clinical experience. The others were faculty and graduate students (Table 1). 

**Table 1:** Participant demographics (N = 63)

Variable	Category	n (%)
Sex	Male	24 (38.1)
	Female	39 (61.9)
Profession	Nurse	45 (71.4)
	Doctor	7 (11.1)
	Teacher	6 (9.5)
	Graduate student	2 (3.2)
	Others	3 (4.8)
Experience	1-5 years	10 (15.9)
	6-10 years	13 (20.6)
	11-15 years	12 (19.0)
	Over 16 years	25 (39.7)
	Anonymous	3 (4.8)

Levels of satisfaction with the study sessions

**Figure 1:**
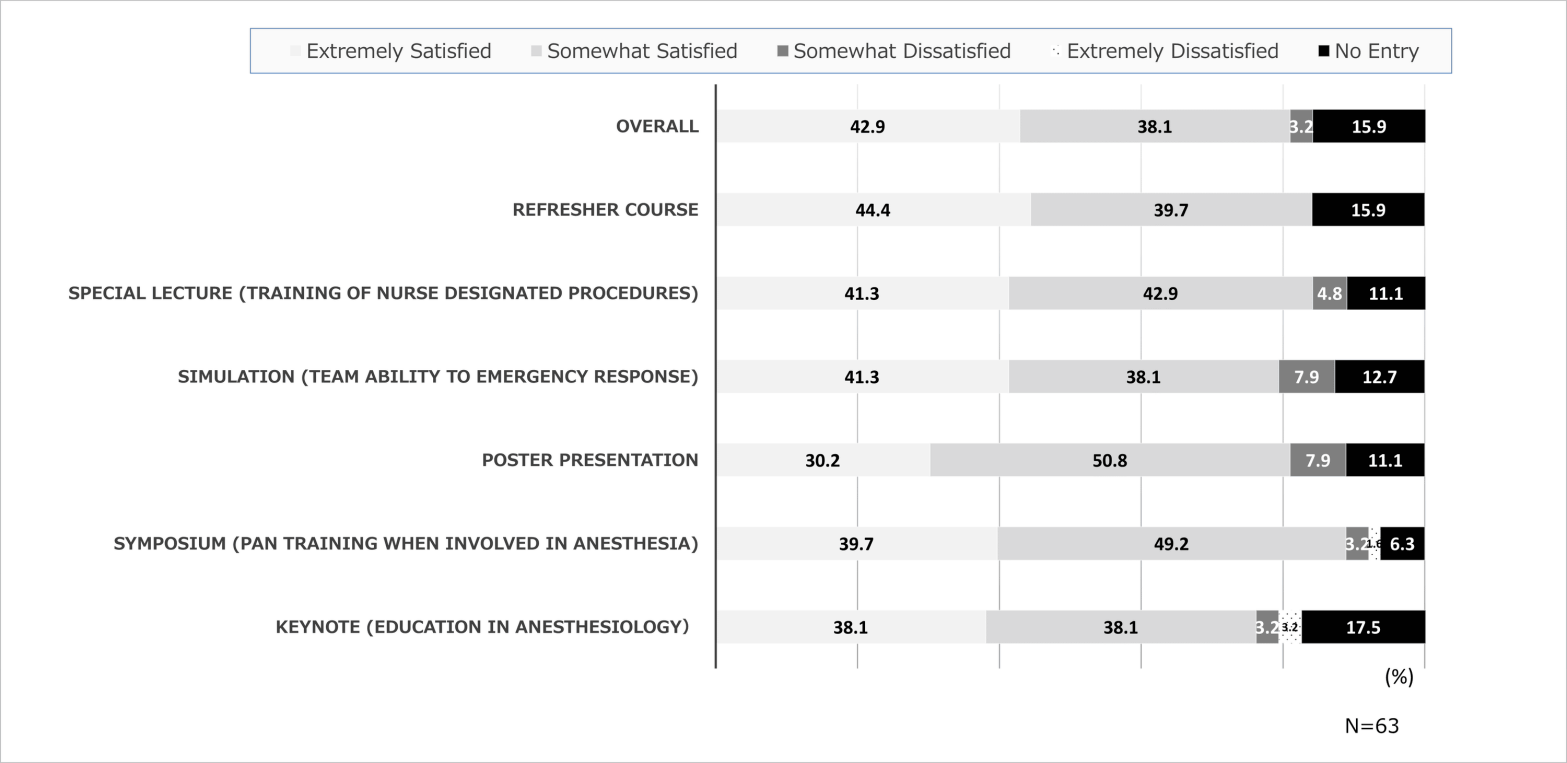
Satisfaction levels with the study sessions

The results of the comprehensive evaluation for the study sessions showed that 51 participants (81.0%) were “extremely satisfied” or “somewhat satisfied,” while only 2 (3.2%) were “extremely dissatisfied” or “somewhat dissatisfied” (Figure 1, Table 2). The keynote lecture, anesthesiology education, had a high level of satisfaction (n = 52, r = 0.74), but the special lecture, a training for nurse-designated procedures, had the lowest level of satisfaction (n = 56, r = 0.51). 

**Table 2:** Satisfaction with the study sessions **p < 0.01. r: Spearman rank correlation coefficient. Correlation of individual sessions with total program satisfaction.

Contents	r	p-value
Keynote (education in anesthesiology）	0.74	**
Symposium (PAN training when involved in anesthesia)	0.58	**
Poster presentation	0.56	**
Simulation (pediatric anesthesia: team's ability in emergency response)	0.55	**
Special lecture (training of nurse designated procedures)	0.51	**
Refresher course	0.65	**

**Figure 2:**
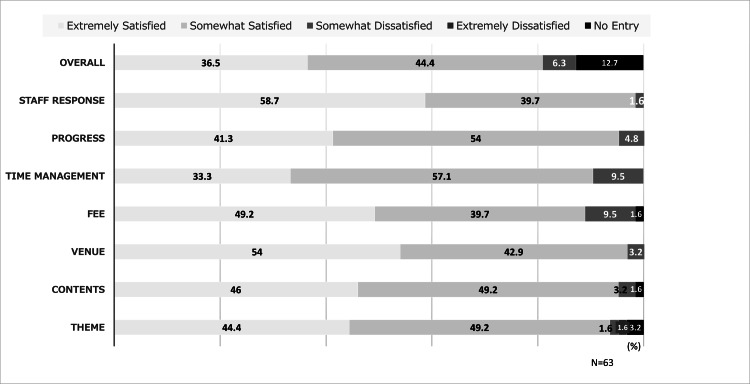
Satisfaction levels with planning and operation

The results of the comprehensive evaluation for planning and operation showed that 50 participants (80.9%) were “extremely satisfied” or “somewhat satisfied,” while only a small minority were “extremely dissatisfied” or “somewhat dissatisfied” (Figure 2, Table 3). The progress had a high level of satisfaction (n = 63, r = 0.76), but the time management had the lowest level of satisfaction (n = 63, r = 0.63). 

**Table 3:** Satisfaction with planning and operation **p < 0.01. r: Spearman rank correlation coefficient. Correlation of individual sessions with total program satisfaction.

Contents	r	p-value
Theme	0.67	**
Contents	0.72	**
Venue	0.72	**
Fee	0.66	**
Time management	0.63	**
Progress	0.76	**
Staff response	0.69	**

Categorization of participants’ comments

The comments, which contain feedback and future requests of the study session, were predominantly related to participants’ recommendations for a training program, in addition to PANs’ activities (Table 4). Twelve codes were extracted from the comments, and three subcategories/categories were determined. The three categories were “Suggestions for a project of multidisciplinary exchange meeting,” “Lack of understanding of PAN’s activities,” and “Questions about the significance of PAN’s existence.” 

**Table 4:** Categorization of participants’ comments

Main categories	Subcategories	Example of codes
Suggestions for a project of multidisciplinary exchange meeting	Hopes for discussion meeting and multidisciplinary project	It would be better to hold a discussion meeting, etc., with those involved
		It would be better to collaborate with other academic societies or associations
		I hope to continue a study session on themes relevant to education
		What is my role if I collaborate with PAN?
Lack of understanding of PAN’s activities	Requests for understanding of PAN’s activity and current situation	I want to know more about the policy of the study sessions
		I want to know more about the contents and reality of activities
		I want to know about the educational contents offered by each training facility
		How much have the expected roles been carried out?
		I want to know nurses’ activities
Questions about the significance of PAN’s existence	Questions on why PANs say nothing about their own work	PANs say nothing about their own activities or future visions
		All lectures were given by physicians. Whose study session is this?
		I felt that PANs did not actively join a discussion

## Discussion

Nursing roles and specialization in the emerging field of perianesthesia care

The present study revealed high levels of satisfaction among participants in the perianesthesia nursing study meeting. This finding suggests that continuing education programs addressing perioperative care are highly valued by healthcare professionals. Continuing professional education plays a critical role in maintaining competence and enhancing motivation among healthcare providers. However, qualitative findings also revealed important challenges related to the recognition of the PAN role. Participants indicated limited understanding of PAN activities and questioned the professional significance of this emerging role [6]. Such concerns are commonly observed during the early stages of professionalization in emerging healthcare specialties.

Internationally, advanced practice nurses have long played key roles in anesthesia-related care. In the United States, CRNAs have been providing anesthesia services for more than a century and currently constitute a substantial portion of the anesthesia workforce. Studies have demonstrated that anesthesia care provided by nurse anesthetists is safe and cost-effective [1,4,7]. In addition to CRNAs, advanced practice nurses in perioperative settings contribute to patient assessment, postoperative pain management, and perioperative care coordination. These roles highlight the potential contributions of specialized nurses in improving perioperative care quality and efficiency [3,8,9]. Compared with these international models, the development of perianesthesia nursing in Japan is still in an early stage. Educational programs for PANs have been gradually expanding, but the role of PANs within healthcare systems remains under discussion [5,8,10,11].

Despite these challenges, the field of perianesthesia nursing in Japan has progressed steadily in recent years. Educational initiatives and professional networking opportunities, such as study meetings and academic conferences, have contributed to the dissemination of knowledge and the development of professional identity among PANs. Furthermore, the establishment of professional organizations dedicated to perianesthesia nursing has provided a platform for academic exchange and professional development. Such initiatives are expected to enhance recognition of the PAN role and promote interdisciplinary collaboration in perioperative care [5,12].

Future directions

The development of specialized nursing roles in perioperative care may help address challenges related to increasing surgical demand and workforce shortages [1,13]. Advanced practice nurses can contribute to patient safety, quality improvement, and team-based care in perioperative settings [2,5]. Future efforts should focus on clarifying the scope of practice of PANs, strengthening educational programs, and promoting interdisciplinary collaboration [12,14]. While the primary data were collected in 2019 and no subsequent longitudinal survey was conducted, this dataset has acquired renewed significance. The rapid evolution of nursing roles, driven by national labor reforms and the acceleration of task-shifting from physicians, makes the findings of this survey critically relevant to the current discourse on nursing professionalization in Japan. Longitudinal research examining the outcomes of perianesthesia nursing education and clinical practice will also be essential for establishing evidence-based practice in this emerging field.

Perianesthesia nursing is a relatively new specialty in Japan, but internationally, the nurse anesthetist and advanced practice nurse roles have developed in diverse ways [10]. Literature indicates that the scope of practice and autonomy of nurse anesthetists vary by country. For example, US CRNAs complete master’s‑level education and practice independently, planning and delivering anesthesia care and pain management. Countries such as the United States, Sweden, France, and Switzerland have well‑established, autonomous nurse anesthetist roles, whereas in Finland, nurse anesthetists typically hold bachelor’s‑level education and work under physician supervision, and Austria is still developing its advanced practice framework. Nonetheless, the common direction across countries is toward advanced practice expertise: high-level clinical judgment and specialized competence.

In light of these international trends, PANs in Japan currently function mainly as collaborative assistants to anesthesiologists, aligning with a safety‑oriented regulatory framework but not yet meeting the autonomy expected of a distinct profession. Research on introducing advanced practice nurses has shown benefits, such as improved patient satisfaction, better retention of nurses, and enhanced management of critically ill patients [1]. Developing a robust career pathway for PANs is therefore urgent [14]. Japanese PANs must redefine their role to establish a level of professional autonomy comparable to that of nurse practitioners and clinical nurse specialists, which will require higher‑level education and legal and institutional support for an expanded scope of practice [15,16].

Limitations

This study has several limitations. First, the survey sample was limited to participants of a single study session and included only 63 respondents, so generalizability is restricted. Second, satisfaction ratings were based on self‑reports and may be influenced by social desirability bias. Third, the data were cross‑sectional; we could not assess the long‑term effects of PAN education. Finally, while this discussion draws on international literature, differences in healthcare systems and cultural contexts mean that direct comparisons may be difficult. Future research should include larger, multi-site samples and longitudinal designs to more fully evaluate PAN professional development and educational outcomes.

## Conclusions

This survey of a perianesthesia nursing continuing education program found high satisfaction among participants, most of whom were experienced nurses. The majority of participants rated the educational sessions and program management positively, with the keynote lecture on anesthesiology education receiving the highest evaluation. Nevertheless, some participants expressed dissatisfaction with time allocation and showed limited understanding of the PANs' role. Content analysis of free comments revealed desires for more multidisciplinary exchange, better visibility of PAN activities, and clarification of the profession’s significance.

The broader context shows that nurse anesthetists around the world practice under very different conditions, ranging from autonomous advanced practitioners to assistants working under physician supervision, yet the global trajectory is toward higher levels of clinical expertise and professional independence. Japan’s current collaborative model ensures safety but lacks the autonomy characteristic of advanced practice nursing. For PANs to contribute fully to perioperative care and align with international standards, their role and educational pathway need to be redefined, and legal and institutional frameworks must evolve to support a greater scope of practice.

## Appendices

**Table 5:** Survey questionnaire

Survey questionnaire	
Q1. Gender	
Male	
Female	
Prefer not to say	
Q2. Current professional role	
Nurse	
Physician	
Allied health professional	
Faculty	
Student	
Other (please specify: ______)	
Q3. Years of experience in your current profession	
1-5 years	
6-10 years	
11-15 years	
16 years or more	
Q4. How did you learn about this conference?	
Conference flyer	
Bulletin board/poster	
Website	
Email	
Word of mouth	
Other (please specify: ______)	
Q5. Satisfaction with conference planning and management	
Please rate your level of satisfaction with each of the following aspects:	
(1 = very satisfied; 2 = satisfied; 3 = dissatisfied; 4 = very dissatisfied)	
Item	Rating
5-1. Theme	1 / 2 / 3 / 4
5-2. Content	1 / 2 / 3 / 4
5-3. Venue	1 / 2 / 3 / 4
5-4. Registration fee	1 / 2 / 3 / 4
5-5. Time allocation	1 / 2 / 3 / 4
5-6. Smoothness of program operation	1 / 2 / 3 / 4
5-7. Staff responsiveness	1 / 2 / 3 / 4
5-8. Networking session	1 / 2 / 3 / 4
5-9. Overall evaluation of planning and management	1 / 2 / 3 / 4
Free comments: Please provide any specific feedback regarding the above items.	
Q6. Satisfaction with program content	
Please rate your level of satisfaction with each of the following:	
(1 = very satisfied; 2 = satisfied; 3 = dissatisfied; 4 = very dissatisfied)	
Item	Rating
6-1. Keynote lecture	1 / 2 / 3 / 4
6-2. Panel discussion	1 / 2 / 3 / 4
6-3. E-poster presentations	1 / 2 / 3 / 4
6-4. Simulation demonstration	1 / 2 / 3 / 4
6-5. Special lecture	1 / 2 / 3 / 4
6-6. Refresher course	1 / 2 / 3 / 4
6-7. Overall evaluation of program content	1 / 2 / 3 / 4
Free comments: Please provide any specific feedback regarding the program content.	
